# Porous Pt–NiO_*x*_ nanostructures with ultrasmall building blocks and enhanced electrocatalytic activity for the ethanol oxidation reaction†

**DOI:** 10.1039/c7ra11575j

**Published:** 2018-01-02

**Authors:** Bangquan Li, Hongsheng Fan, Ming Cheng, Yuanjun Song, Fangtao Li, Xiaodan Wang, Rongming Wang

**Affiliations:** Department of Physics, Beihang University Beijing 100191 China; Institute of Solid State Physics, Shanxi Datong University Datong 037009 China; Beijing Key Laboratory for Magneto-Photoelectrical Composite and Interface Science, School of Mathematics and Physics, University of Science and Technology Beijing Beijing 100083 China rmwang@ustb.edu.cn

## Abstract

Oxidized species on surfaces would significantly improve the electrocatalytic activity of Pt-based materials. Constructing three-dimensional porous structures would endow the catalysts with good stability. Here, we report a simple strategy to synthesize porous Pt–NiO_*x*_ nanostructures composed of ultrasmall (about 3.0 nm) building blocks in an ethanol–water solvent. Structure and component analysis revealed that the as-prepared material consisted of interconnected Pt nanocrystals and amorphous NiO_*x*_ species. The formation mechanism investigation revealed that the preformed amorphous compounds were vital for the construction of porous structure. In the ethanol oxidation reaction, Pt–NiO_*x*_/C exhibited current densities of 0.50 mA cm_Pt_^−2^ at 0.45 V (*vs.* SCE), which were 16.7 times higher than that of a commercial Pt/C catalyst. Potentiostatic tests showed that Pt–NiO_*x*_/C had much higher current and better tolerance towards CO poisoning than the Pt/C catalyst under 0.45 V (*vs.* SCE). In addition, the NiO_*x*_ species on the surface also outperformed an alloyed Ni component in the test. These results indicate that the Pt–NiO_*x*_ porous nanomaterial is promising for use in direct ethanol fuel cells.

## Abstract 2

A porous Pt–NiO_*x*_ nanomaterial was constructed by a simple strategy to achieve excellent ethanol oxidation catalyst performance.

## Graphical abstract

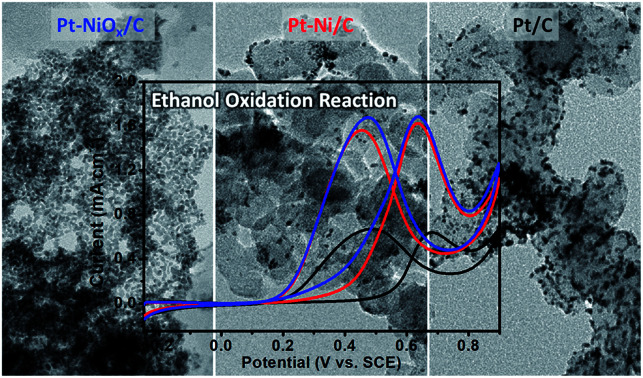

## Introduction

**Scheme 1:**
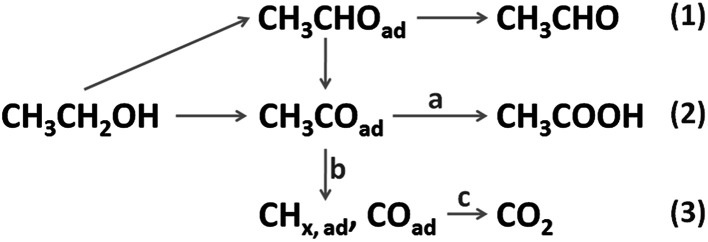
Ethanol oxidation routes on Pt-based catalyst.

Fuel cells fed with hydrogen or small organic molecules have shown great prospects in transportation and stationary power supplies, with their merits of high energy conversion efficiency, sustainable fuel sources and low harmful gas emissions.^1,2^ As a common liquid fuel, ethanol can be easily handled, stored and transported, with a high energy density (8 kW h kg^−1^, 6.32 kW h L^−1^) and a low toxicity. Its production is technologically convenient and economic from various agricultural products and biomass.^3^ Therefore, the direct ethanol fuel cell (DEFC) is regarded as one of the most promising energy sources. The process of the ethanol oxidation reaction (EOR) is quite complicated on a real electrolyte–electrode interface under an applied potential, which increases the difficulty in understanding the reaction mechanism. On the basis of relevant literature,^4^ the possible EOR routes are briefly depicted in Scheme 1. As shown, there are three final products, that is, CH_3_CHO (acetaldehyde), CH_3_COOH (acetic acid) and CO_2_, corresponding to two-, four-, and twelve-electron transfer. Among these routes, complete oxidation of ethanol to CO_2_ is preferred because it offers the highest faradaic efficiency. But in the anode loaded with pure Pt catalysts, the current density is too low and the charge is mainly (usually >90%) generated from incomplete oxidation reactions.^5^ According to previous studies,^4–6^ several intrinsic reasons are responsible for these drawbacks: the cleavage of the C–C bond is necessary for CO_2_ generation (reaction b), but this process is kinetically sluggish on pure Pt surfaces; the carbonous intermediates (CO, CH_*x*_, *etc.*) are generated and even a small amount of them would block the active sites of the Pt catalyst severely, which hampers the dissociative adsorption of ethanol; the adsorbed amount of oxygenated species (OH and O) is low on the pure Pt surface at a small overpotential, thus slowing the surface reactivation process (reaction a and c) down. Since Pt is generally regarded as the best monometallic EOR catalyst, the above-mentioned drawbacks severely hinder the practical application and commercialization of DEFC technology.

In recent years, great efforts have been made to explore the EOR mechanism on Pt-based catalysts and gain a considerable oxidation current density. The most studied strategy is integrating oxophilic metals with Pt to form alloy nanostructure. For instance, bimetallic Pt–Sn,^7^ Pt–Co^8^ and trimetallic Pt–Rh–Sn^9^ alloys were reported to present much higher EOR catalytic activity than the Pt/C catalyst. The additive elements favor the adsorption of oxygenated species at a lower potential, and could alter the electronic structure of surface Pt atoms, resulting in a weaker binding of carbonous intermediates. However, investigation of reaction product distribution showed that the addition of Sn and Co would debase the CO_2_ selectivity and increase the product ratio of CH_3_COOH.^8,10^ Recently, Pt (111)–SnO_*x*_,^11^ Pt–SnO_2_ (ref. 12 and 13) and PtRhO_*x*_–SnO_2_ (ref. 14) structures were found to effectively boost the C–C cleavage rate and elevate the total oxidation current density. In these reports, oxidized species on catalyst surface played a vital role. Meanwhile, to avoid the aggregation of ultrasmall (typically <5 nm) nanoparticles, considerable work has been done to construct three-dimensional (3D) interconnected structures, including nanoframes,^15–17^ aerogels,^18–20^ hollow spheres,^21–24^ nanoflowers,^25^ and so on. Rational design and preparation of these self-supported structures are significant for material engineering and practical application.

Nickel and its compounds have been proved to remarkably promote the catalytic activity of Pt in oxidation reactions of CO and methanol.^26–30^ Most recently, octahedral Pt–Ni/C alloying catalyst was found to exhibit a much higher EOR current density than Pt–Ni/C and Pt/C.^31^ In this work, 3D Pt–NiO_*x*_ porous nanostructure was constructed to evaluate the effect of NiO_*x*_ species in the EOR process. The structure, component, morphology and surface chemical state were characterized and analyzed by corresponding technologies. Then, the formation mechanism was investigated by conducting contrast experiments. Finally, the catalytic property for EOR was studied by potentiodynamic and potentiostatic techniques compared with Pt–Ni/C alloy structure and commercial Pt/C catalyst.

## Experimental

The procedure of synthesizing Pt–NiO_*x*_/C is described as follows: (1) NaBH_4_ (12 mg) and VXC-72R carbon (4 mg) were dissolved in ethanol (30 mL in a beaker), and ultrasonic treatment was conducted for 5 minutes; (2) the solution was kept uniform with magnetic stirring (about 600 rpm), and an aqueous solution (1 mL) of NiCl_2_·6H_2_O (5 mg) was added dropwise to the ethanol solution; (3) after 5 minutes, an aqueous solution (2 mL) of K_2_PtCl_6_ (5 mg) was added dropwise; (4) after 30 minutes, the magnetic stirring was turned off; (5) after 6 hours, the supernatant was removed, and then high purity water (30 mL) was added in the beaker with magnetic stirring (about 600 rpm); (6) after 2 hours, the product was collected by centrifugation and washed for several times by ethanol and water, and then put in a vacuum drying oven at 80 °C for 24 hours.

The commercial Pt/C catalyst (40 wt% Pt) was purchased from Alfa Aesar and briefly described as Pt/C.

The structures and morphologies were characterized by X-ray diffraction (XRD; Rigaku, Ultima IV, Cu Kα) and transmission electron microscopy (TEM; JEOL, JEM-2200FS). Elemental composition data were determined by energy-dispersive X-ray spectroscopy (EDS; Oxford, X-Max80T) fitted in the transmission electron microscope (Titan, FEI). The surface chemical states of elements were measured by X-ray photoelectron spectroscopy (XPS; Thermofisher, ESCALAB 250 system, AI Kα).

The electrochemical measurements were carried out with a CHI660D electrochemical workstation (CH Instruments). A glassy carbon disk (*ϕ* = 3 mm), a platinum wire and a saturated calomel electrode (SCE) were used as working, counter, and reference electrodes, respectively. To prepare the working electrode, 4 μL of the sample suspension (about 0.5 mg_Pt_ mL^−1^ in ethanol) was pipetted onto the glassy carbon substrate. After drying in air, a Nafion solution (2.5 μL, 5 wt%) was used to cover the surface of the catalyst electrode. Before tests, the electrolyte solution was bubbled in nitrogen for 60 minutes. The samples were first treated by cyclic voltammetry (CV) in acidic solution for twenty cycles. All of the CV tests were conducted at a sweep rate of 50 mV s^−1^. For CO stripping measurements, CO was absorbed onto the catalysts surface by bubbling high-purity CO through 0.5 M H_2_SO_4_, while the potential scanned from −0.1 to 0.1 V with a rate of 50 mV s^−1^. After the adsorption of CO reached saturation, the dissolved CO was removed from the solution by bubbling high-purity N_2_.

## Results and discussion

### Morphological and structural characterization

The component information was first determined by EDS and the result is presented in Fig. S1.† Several elements were detected, including Pt, Ni, O, C and Cu. The signals of Pt and Ni were ascribed to the reaction resultants of K_2_PtCl_6_ and NiCl_2_·6H_2_O, while those of C and Cu resulted from carbon black and copper grid. The peak of O was attributed to oxidized species which were easily formed in the ethanol–water mixed solution. Based on signal intensity, the mole ratio of Pt and Ni was determined to be 88 : 12. Considering the feed ratio of Pt and Ni was 33 : 67, one could conclude that most of the Ni species had been removed from the final product. In addition, the weight contents of Pt and NiO_*x*_ were about 34% and 3% respectively in the product.

**Fig. 1:**
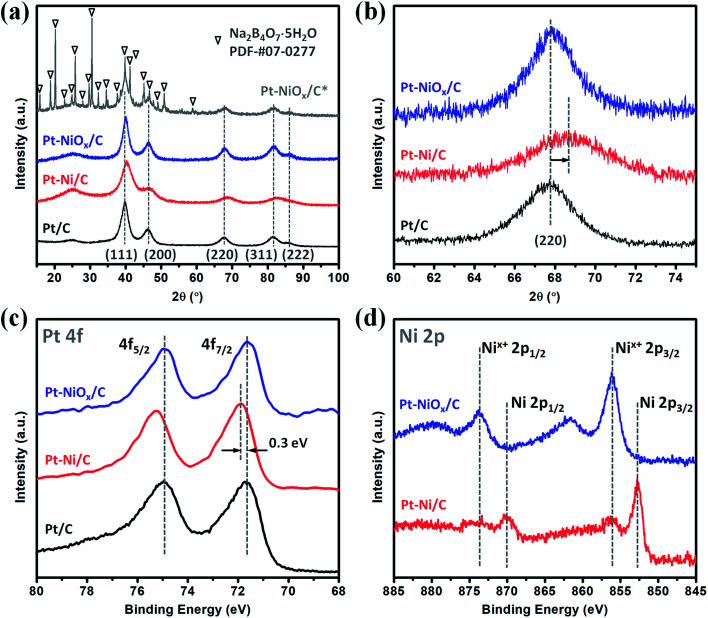
(a) XRD patterns and (b) the corresponding (220) diffraction peaks of Pt–NiO_*x*_/C* (before washing), Pt–NiO_*x*_/C (after washing), Pt–Ni/C and Pt/C. XPS spectra of (c) Pt 4f and (d) Ni 2p core levels of Pt–NiO_*x*_/C, Pt–Ni/C and Pt/C.

The XRD patterns are displayed in Fig. 1(a) for the as-prepared samples (Pt–NiO_*x*_/C*, precipitate collected before washing; Pt–NiO_*x*_/C, final product). The final product demonstrated diffraction pattern of face-centered cubic (fcc) Pt structure, which was similar to that of Pt/C. Besides the diffraction pattern of Pt structure, Pt–NiO_*x*_/C* also exhibited a series of much sharper diffraction peaks corresponding to tincalconite (Na_2_B_4_O_7_·5H_2_O). This by-product was ascribed to the oxidation of NaBH_4_ in ethanol–water mixed solution containing metal salt precursors. The rapid reaction of NaBH_4_ generated amorphous boron-based compounds, which were hardly dissolved in ethanol and could not be washed away by ethanol. After dried under vacuum condition at 80 °C, these compounds crystallized to form tincalconite. Although the EDS data revealed that the mole ratio of Pt and Ni was 88 : 12 in the final product, the XRD patterns showed no crystalline Ni-based component or peak shift relative to Pt/C (as shown in Fig. 1(b)), indicating that Ni atoms existed in amorphous Ni-based oxidized species (labeled as NiO_*x*_). Using the Debye–Scherer formula, the average sizes of crystalline grains of Pt were calculated to be about 3 nm for both Pt–NiO_*x*_/C and Pt/C. In addition, the broadened peak at about 25° corresponded to the (002) plane of carbon black with hexagonal structure.^32^

The XPS spectra of Pt 4f and Ni 2p core levels are demonstrated in Fig. 1(c) and (d). Pt–NiO_*x*_/C exhibited similar Pt 4f binding energies (71.6 eV for 4f_7/2_; 74.9 eV for 4f_5/2_) to Pt/C, confirming the pure Pt phase. And its Ni 2p spectra showed only oxidized Ni signal at about 856.1 eV for 2p_3/2_ and 873.7 eV for 2p_1/2_.^33,34^

**Fig. 2:**
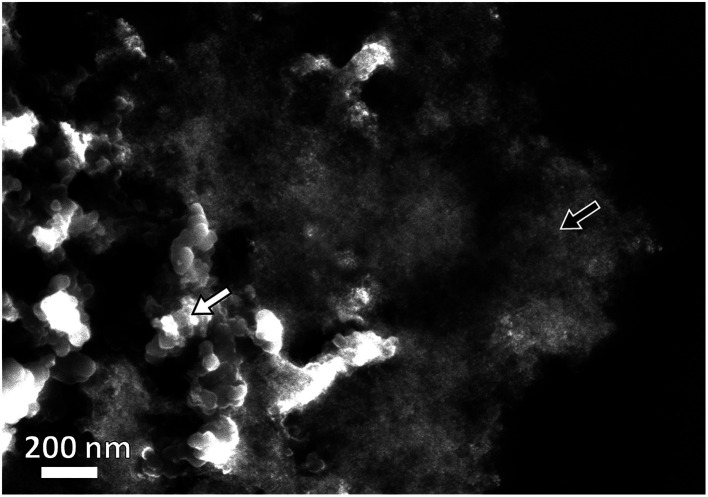
Representative SEM image of Pt–NiO_*x*_/C. The black arrow shows Pt–NiO_*x*_ and the white one shows carbon black particles.

**Fig. 3:**
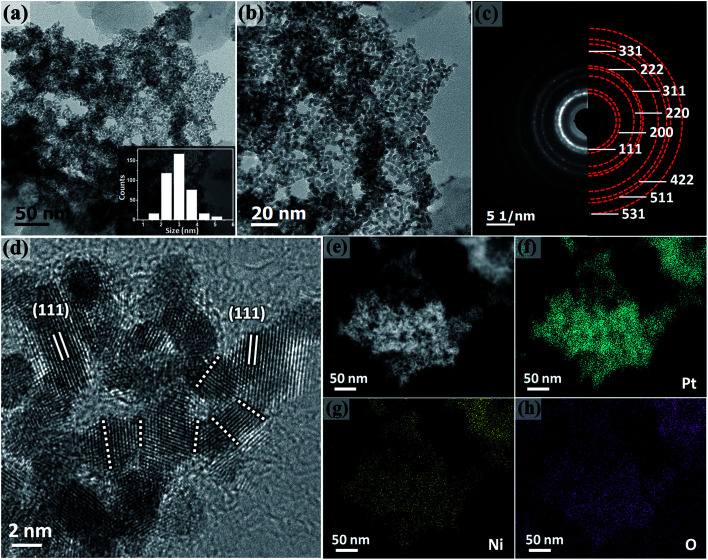
(a and b) Representative low-magnification TEM images of Pt–NiO_*x*_/C. The inset in (a) is the particle size distribution histogram of Pt–NiO_*x*_/C. (c) SAED pattern of Pt–NiO_*x*_/C. (d) HRTEM image of Pt–NiO_*x*_/C. The dotted lines demonstrate crystal boundaries. (e) HAADF image of Pt–NiO_*x*_/C; (f–h) corresponding component mapping of Pt, Ni and O elements.

The morphology of Pt–NiO_*x*_/C was first characterized by SEM with a typical image exhibited in Fig. 2. The black arrow showed 3D structure with a size of about 1 μm assembled by small building blocks, while the white arrow pointed out carbon black particle with relatively smooth surface. TEM technique was employed to acquire more information. Low-magnification images in Fig. 3(a) and (b) exhibited porous nanostructure with interconnected building blocks. The particle sizes were measured in the TEM images based on 400 randomly selected particles and the histogram was inserted in Fig. 3(a). The average size was about 3.0 nm with a standard deviation of about 0.7 nm in consistence with the value derived from XRD data. Selected area electron diffraction (SAED) gave an annular fcc diffraction pattern in Fig. 3(c), indicating polycrystalline feature of the 3D structure. Generally, discrete nanoparticles tend to aggregate in the absence of capping agent or surfactant to reduce the total surface energy, especially for catalyst nanoparticles with sizes less than 10 nm.^35^ Constructing 3D porous structure can effectively prevent such aggregation, and moreover, the confined space is beneficial for the formation of heterogeneous interface between amorphous NiO_*x*_ species and Pt building blocks. The high-resolution TEM (HRTEM) images in Fig. 3(d) provided lattice-resolved information. Distinct lattice fringes corresponding to Pt (111) plane were easily found with random orientations, and this again confirmed the polycrystalline structure. Lattice dislocation and crystal boundary were observed as marked in Fig. 3(d), which resulted from rapid deposition of Pt atoms and growth of crystals in NaBH_4_ solution.

Component mapping was conducted to determine the distribution of Pt and Ni elements. In the high angle annular dark field (HAADF) images in Fig. 3(e), bright white regions corresponded to the existence of Pt-based or Ni-based materials. In the mapping results presented in Fig. 3(f–h), Pt–NiO_*x*_/C showed uniform distribution of Pt, Ni and O elements in the material regions determined by HAADF images, indicating that NiO_*x*_ species were evenly mixed with interconnected Pt nanocrystals.

### Formation mechanism

**Fig. 4:**
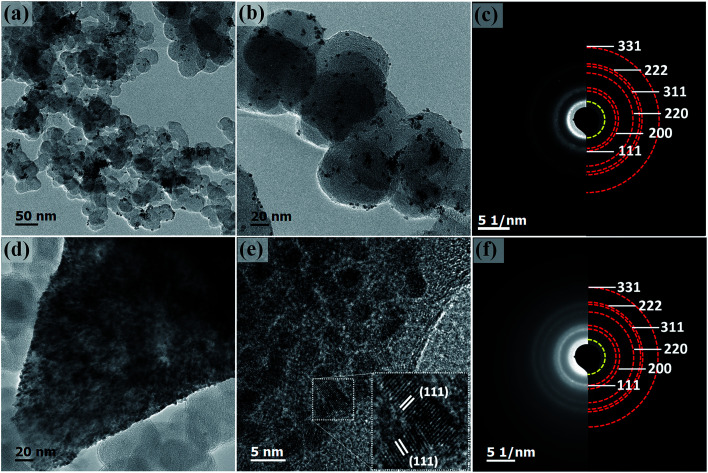
Representative TEM images: (a and b) Pt–Ni/C, and (d and e) Pt–NiO_*x*_/C* (before washing); (c and f) corresponding SAED patterns. The red and yellow semi-circles in (c) and (f) show diffraction rings of fcc crystalline structure and carbon black respectively. The inset in (e) is an enlarged image showing lattice-resolved information.

**Fig. 5:**
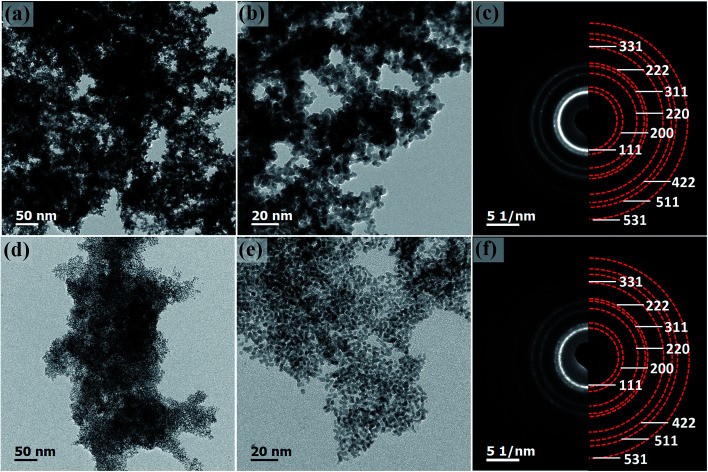
Representative TEM images: (a and b) Pt–NiO_*x*_ aggregated structure, and (d and e) Pt–NiO_*x*_ porous structure without carbon black; (c and f) corresponding SAED patterns. The red semi-circles in (c and f) show diffraction rings of fcc crystalline structure.

To investigate the formation mechanism, a series of contrast experiments were carried out. While K_2_PtCl_6_ was added as the only metal salt precursor, the generated Pt nanoparticles were dispersed quite evenly on carbon black as shown in Fig. S2.† Similarly, when K_2_PtCl_6_ was first mixed with NiCl_2_·6H_2_O, the resultant nanoparticles were discretely adsorbed on carbon black as shown in Fig. 4(a) and (b). SAED pattern in Fig. 4(c) showed an fcc structure and polycrystalline feature, and the inner diffraction ring was attributed to carbon black. The fcc diffraction rings of Pt–Ni/C were slightly larger than those of Pt–NiO_*x*_/C as shown in Fig. S3,† indicating a smaller lattice constant due to alloying effect of Pt and Ni. EDS spectrum was presented in Fig. S4,† and the mole ratio of Pt and Ni was determined to be 86 : 14. XRD pattern in Fig. 1(a) and (b) also exhibited alloy feature of Pt–Ni/C, that is, the positive shift of peak position. The mole ratio of Pt : Ni could be calculated to be 86 : 14, which matched with the value derived from EDS data. In Fig. 1(c), the binding energy of Pt 4f core level showed a positive shift of about 0.3 eV relative to that of Pt/C. The XPS spectrum of Pt–Ni/C in Fig. 1(d) showed strong metallic Ni signal at about 852.7 eV for 2p_3/2_ and 873.7 eV for 2p_1/2_. Here, the positive shift of Pt 4f core levels in Pt–Ni/C could be ascribed to the alloying effect of Pt and Ni. According to a previous report,^36^ the electron transfer occurs from Ni to Pt in the Pt–Ni alloy structure, which results in change of work function and upshift of the reference level (*E*_F_) in photoelectron measurements. Therefore, the Pt 4f core level equivalently shifts in an opposite direction, leading to a higher binding energy. Based on the above analysis, this sample was denoted as Pt–Ni/C. In summary, these two experimental results indicated that adding NiCl_2_·6H_2_O in advance was benefit for the formation of 3D structure. This might be associated with the unique role of amorphous Ni–B compounds in the ethanol–water solvent. According to our previous work,^23,29^ amorphous Ni–B compounds were generated with the reduction of NiCl_2_ by NaBH_4_. Compared with carbon black, these compounds might have stronger adsorption capacity for Pt seeds, which resulted in high-density deposition of Pt seeds on Ni–B compound agglomerate. Then, oriented crystal growth promoted the connection of discrete Pt seeds, and the continuously generated boron-based compounds would meanwhile prevent the agglomeration of nanocrystals. In the oxygen-enrichment environment, metallic Ni elements were prone to be oxidized, leading to the formation of Pt–NiO_*x*_. When fully washed with water, some resultants were removed, including water soluble boron-based compounds and most amorphous Ni-based species. NiO_*x*_ species maintained as interfacial structure due to their strong interaction with porous Pt structure. A representative TEM image of Pt–NiO_*x*_/C* shown in Fig. 4(d) and (e) confirmed this speculation. Low-magnification TEM image showed that high-density nanoparticles were supported on a bulk material. SAED pattern in Fig. 4(f) exhibited fcc diffraction rings of Pt polycrystalline structure and a dispersive circular background corresponding to amorphous structure. HRTEM image showed that the nanoparticles were crystallized with distinct Pt (111) lattice fringes, while the bulk supporter exhibited amorphous feature. In addition, carbon black improves the dispersion effect of Ni–B compounds, since the former has better dispersion capacity in ethanol–water solvent and could alleviate the aggregation of resultants. Without addition of carbon black, severely aggregated material with fcc structure was achieved as shown in Fig. 5(a–c). Adding long-chained surfactant could also prevent the aggregation effect. When carbon black was replaced with 50 mg PVP, 3D porous structure was successfully synthesized as shown in Fig. 5(d–f).

### Electrocatalytic properties

**Fig. 6:**
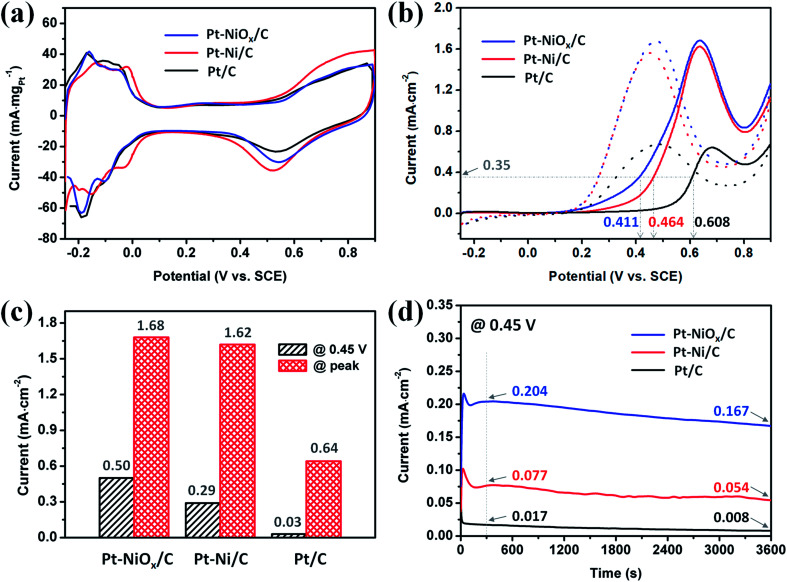
Electrochemical measurements of Pt–NiO_*x*_/C, Pt–Ni/C and Pt/C at 25 ± 1 °C. (a) CV curves for calculating ECSA in an aqueous solution of 0.5 M H_2_SO_4_. (b) ECSA-normalized CV curves in an aqueous solution of 1 M ethanol and 0.5 M H_2_SO_4_. (c) Oxidation current at 0.45 V and at peak position in (b). (d) ECSA-normalized CA curves in an aqueous solution of 1 M ethanol and 0.5 M H_2_SO_4_ under 0.45 V.

The CV curves obtained in acidic solution are depicted in Fig. 6(a). Apparent hydrogen adsorption and desorption region could be distinguished in the range of −0.25 to 0.1 V. And the electronic double layer region appeared in the range of about 0.1 to 0.35 V. At a potential higher than 0.35 V, the formation of Pt–O species and their reduction were observed with current increasing during positive-going scan and a current peak during negative-going scan. The electrochemical active surface areas (ECSAs) were determined based on the hydrogen desorption region after deduction of the double-layer charge. The ECSAs of Pt–NiO_*x*_/C, Pt–Ni/C and Pt/C were calculated to be 58.6, 60.5 and 67.3 m^2^ g_Pt_^−1^.

The EOR properties were first measured using the CV method from −0.25 to 0.9 V in an aqueous solution of 1 M ethanol and 0.5 M H_2_SO_4_ at 25 ± 1 °C. The complete curves are presented in Fig. 6(b), and the oxidation currents were normalized to the corresponding ECSAs. All of the currents underwent an increment from 0.1 V to a specific potential between 0.6 V and 0.7 V, which is attributed to easier dissociation of H_2_O and formation of OH adsorbed species at a higher potential and thus increased electrode reaction kinetics. Then, the current decreased with the scan going because the oxidation of surface Pt atoms competes with dissociative adsorption of CH_3_CH_2_OH. The peak potentials of Pt–NiO_*x*_/C and Pt–Ni/C were smaller than that of Pt/C, indicating the existence of NiO_*x*_ species on surface and alloyed Ni atoms facilitated the oxidation of Pt atoms at a lower potential. On the positive-going scan, the potential required to achieve a current density of 0.35 mA cm^−2^ located at around 0.411 V for Pt–NiO_*x*_/C, which was 53 mV and 197 mV negative to that of Pt–Ni/C and Pt/C, suggesting excellent EOR catalytic kinetics of Pt–NiO_*x*_/C. The current densities at 0.45 V and at peak position are compared in Fig. 6(c). At 0.45 V, Pt–NiO_*x*_/C showed a current density of 0.50 mA cm^−2^, which was 1.7 and 16.7 times higher than that of Pt–Ni/C and Pt/C. At peak position, Pt–NiO_*x*_/C showed a current density of 1.68 mA cm^−2^, which was similar to that of Pt–Ni/C (1.62 mA cm^−2^) and 2.6 times higher than that of Pt/C. From these data, one could conclude that the as-prepared Pt–NiO_*x*_/C demonstrated higher EOR catalytic activity than Pt–Ni/C and Pt/C, especially at a lower potential. The above results suggested that surface oxidized species and atomic configuration played important role in EOR. The steric hindrance of 3D porous structure was benefit for the formation of Pt–NiO_*x*_ mixed structure. According to previous reports,^7–9,11,12,14,37^ surface oxidized species and alloyed component were both able to improve the EOR catalytic activity of Pt. In this work, NiO_*x*_ species on surface might lead to more direct and stronger promoting effect in oxidation of adsorbed carbonous intermediate species than alloyed component, and thus effectively elevate the oxidation current. CO stripping measurement was conducted to evaluate the effect of NiO_*x*_ species and the tolerance of Pt-based catalysts towards CO poisoning. The results were demonstrated in Fig. S5.† The peak potentials were 0.488, 0.600, and 0.685 V for Pt–NiO_*x*_/C, Pt–Ni/C, and Pt/C, respectively. And the onset potentials where the current reached 10% of peak values were 0.449, 0.517, and 0.620 V for Pt–NiO_*x*_/C, Pt–Ni/C, and Pt/C, respectively. Lower peak and onset potentials indicated an easier oxidation of absorbed CO species on Pt surface.^31,38^ For Pt–NiO_*x*_/C, rich oxygenated species were thought to effectively facilitate the oxidation of CO at a low potential. For Pt–Ni/C, the change of Pt electronic structure would result in a weaker CO adsorption strength, and surface Ni atoms could also assist the formation of oxygenated species according to the bifunctional mechanism.^39,40^ Here, Pt–NiO_*x*_/C showed the best CO oxidation property, which confirmed the excellent effect of NiO_*x*_ in preventing CO poisoning on Pt-based catalysts.

The potentiostatic tests were conducted using the chronoamperometry (CA) method at 0.45 V in an aqueous solution of 1 M ethanol and 0.5 M H_2_SO_4_ at 25 ± 1 °C. The curves are displayed in Fig. 6(d). All of the currents underwent a severe decay in the first dozens of seconds due to the generation and adsorption of carbonous intermediates that hindered the catalytic oxidation process on catalyst surface. Then, the currents held a relatively stable status. The current ratio was 12.0 : 4.5 : 1 after 300 s with the sequence of Pt–NiO_*x*_/C, Pt–Ni/C and Pt/C. The currents continuously decreased, and after 3600 s, their current ratio changed to be 20.9 : 6.8 : 1. This result showed that Pt–NiO_*x*_/C owned much better EOR catalytic activity and tolerance towards CO poisoning at 0.45 V than Pt–Ni/C and Pt/C. The excellent property of Pt–NiO_*x*_/C was ascribed to its rich NiO_*x*_ species, which promoted the EOR kinetics and alleviated the poisoning effect of surface active sites resulting from continuous decomposition of CH_3_CH_2_OH.

## Conclusions

Three-dimensional Pt–NiO_*x*_ porous nanostructure was synthesized in ethanol–water solvent at room temperature. Structure and component characterizations demonstrated that the as-synthesized sample was composed of ultrasmall building blocks of about 3.0 nm and amorphous NiO_*x*_ species. Investigation on formation mechanism revealed that the preformed amorphous compounds were vital for the construction of porous structure. Ethanol oxidation reaction was measured employing Pt–NiO_*x*_/C mixed structure, Pt–Ni/C alloy structure and commercial Pt/C catalyst to evaluate their catalytic properties. It was found that NiO_*x*_ species greatly improved the ethanol oxidation catalytic activity and anti-poisoning ability of Pt-based materials, especially at a small overpotential, and this promoting effect was more pronounced than that aroused by Ni alloy component. In general, this work confirms that NiO_*x*_ species on surface and 3D porous structure contributed to the great performance enhancement in ethanol oxidation reaction, and the Pt–NiO_*x*_/C material could be a promising candidate of DEFC catalysts.

## Conflicts of interest

There are no conflicts of interest to declare.

## Supplementary Material

RA-008-C7RA11575J-s001
