# Supplementary material for: Porous Pt–NiOx nanostructures with ultrasmall building blocks and enhanced electrocatalytic activity for the ethanol oxidation reaction
Source: RSC Adv. 2018 Jan 2;8(2):698–705. doi: 10.1039/c7ra11575j (PMC9076817; doi:10.1039/c7ra11575j)
Supplement: RA-008-C7RA11575J-s001 [file RA-008-C7RA11575J-s001.pdf]

Electronic Supplementary Material (ESI) for RSC Advances.
This journal is © The Royal Society of Chemistry 2017

Electronic Supplementary Information

Porous Pt-NiO_x Nanostructure with Ultrasmall Building Blocks and Enhanced Electrocatalytic Activity for Ethanol Oxidation Reaction

Bangquan Li,[a, b] Hongsheng Fan,[a] Ming Cheng,[a] Yuanjun Song,[c] Fangtao Li,[c] Xiaodan Wang,[c] and Rongming Wang*[c]

[a]Department of Physics, Beihang University, Beijing 100191, China

[b]Institute of Solid State Physics, Shanxi Datong University, Datong 037009, China

[c]Beijing Key Laboratory for Magneto-Photoelectrical Composite and Interface Science, School of Mathematics and Physics, University of Science and Technology Beijing, Beijing 100083, China

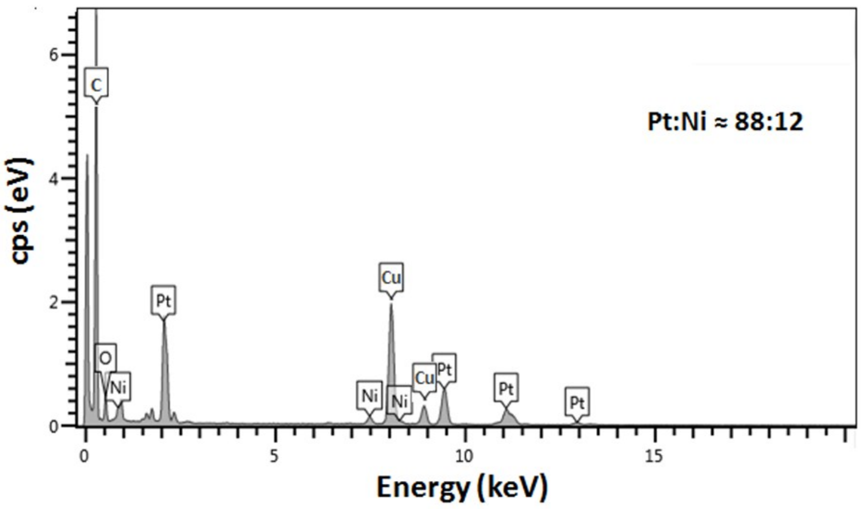

Fig. S1 EDS spectrum of Pt-NiO_x/C.

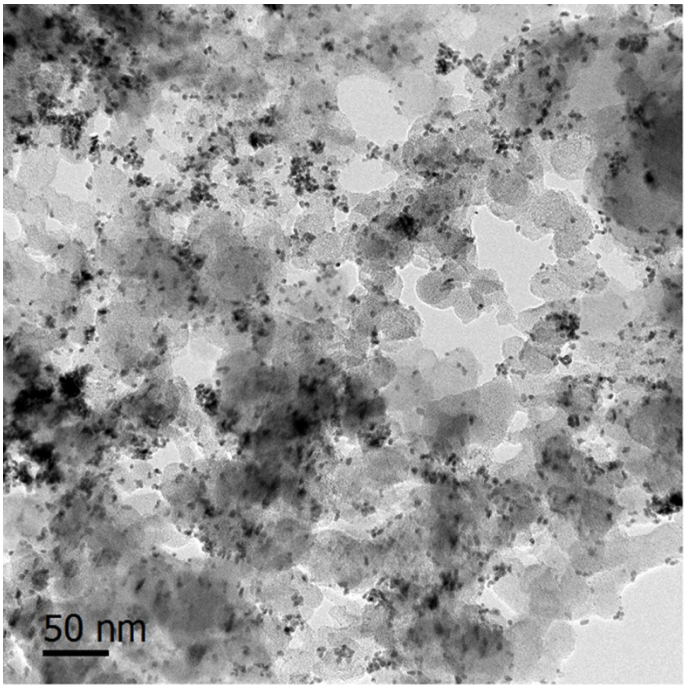

Fig. S2 A representative TEM image of the home-made Pt/C structure.

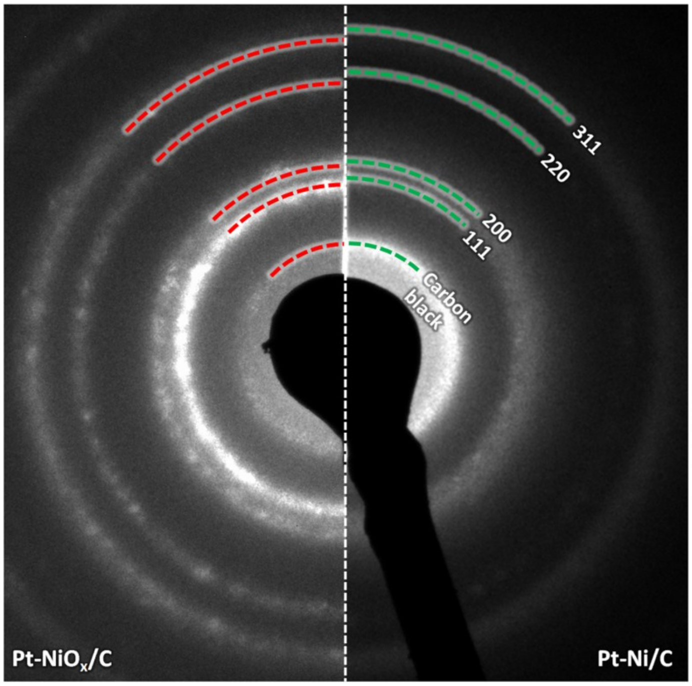

Fig. S3 A comparison of SAED patterns of Pt-NiO_x/C and Pt-Ni/C. The dashed lines show the diffraction rings of carbon black and fcc crystalline structures.

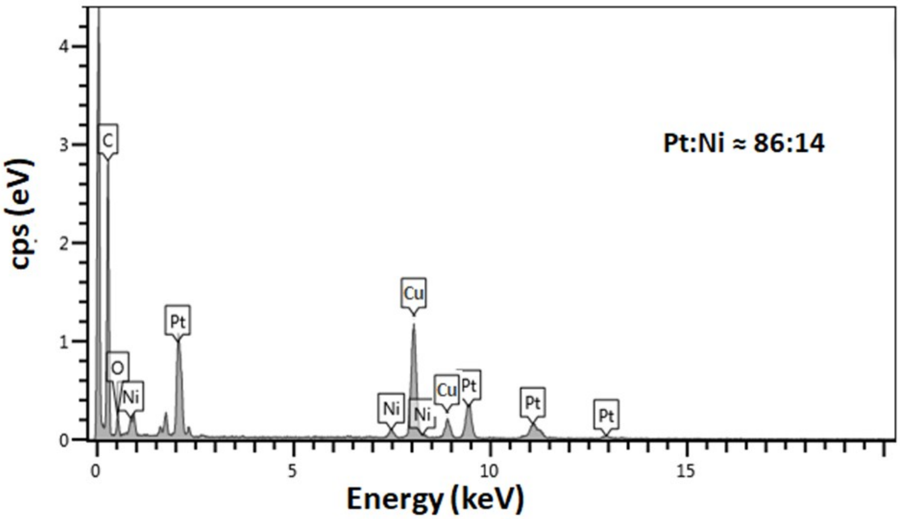

Fig. S4 EDS spectrum of Pt-Ni/C.

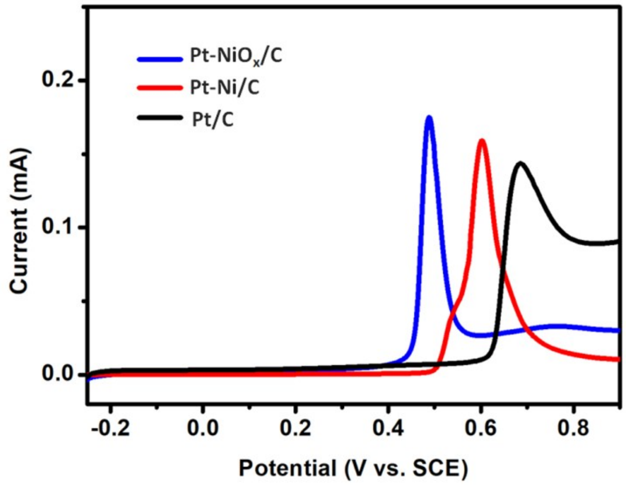

Fig. S5 CO-stripping profiles of Pt-NiO_x/C, Pt-Ni/C and Pt/C catalysts at 20 mV·s^{-1} in an aqueous solution of 0.5 M H_2SO_4.
